# Individual Feed Efficiency Monitoring of Charolaise Candidate Young Bulls in Relation to Feeding Behavior and Self-Performance Test Results

**DOI:** 10.3390/ani12010035

**Published:** 2021-12-24

**Authors:** Gabriella Holló, Henrietta Nagy-Kiszlinger, János Tossenberger, Márton Török, Balázs Húth

**Affiliations:** 1 Kaposvár Campus, Hungarian University of Agriculture and Life Sciences, Guba Sándor Str. 40, H-7400 Kaposvar, Hungary; nagy.kiszlinger.henrietta@uni-mate.hu (H.N.-K.); tossenberger.janos@uni-mate.hu (J.T.); huth.balazs@uni-mate.hu (B.H.); 2 National Association of Hungarian Charolais Cattle Breeders, Vologda u. 3, H-3525 Miskolc, Hungary; torok.marton@charolais.hu

**Keywords:** residual feed intake, Charolaise, candidate bulls, performance test

## Abstract

**Simple Summary:**

Nowadays, in beef cattle breeding programs considerable interest lies in selecting animals that require fewer feed inputs without negatively impacting performance traits, thereby improving the profitability. This study has shown that the feeding behavior of Charolaise candidate young bulls mainly depend on residual feed intake (RFI), whilst RFI had no effect on phenotypic body composition and performance test results (except for feed intake/dry matter intake and gain to feed). Skeletal traits, including back-loin length and rump length as well as frame and muzzle width showed negative associations with RFI value, indicating that the longer, taller, higher frame size bulls were more efficient. At the same time, bulls with better RFI values were associated with forelegs/hind legs weakness. The close relationship of dry matter intake and feed efficiency in Charolaise candidate bulls suggests that measures are relevant and may have an application in the performance test. Performance from individual dry matter intake information may be the most cost-effective way to test a greater number of animals annually.

**Abstract:**

This study evaluated the effect of differences in residual feed intake (RFI) of Charolaise candidate young bulls on feeding behavior and self-performance test results. Bulls were classified into high and low RFI (H-RFI, L-RFI) groups. Bulls were fed in a HOKOFARM system to measure individual animal intake and behavior. L-RFI bulls had significantly lower feed intakes (*p* = 0.002) and higher gain to feed ratio (*p* = 0.001), lower intake per day/kg DM (dry matter) (*p* = 0.002) and lower intake g/body weight/day (*p* < 0.001). L-RFI animals had lower visits number per day (*p* = 0.02), but spent longer time per visit (*p* = 0.02), and tended to have higher intake g/visit (*p* = 0.06) on feeders. The correlation between RFI and DMI (dry matter intake)/bodyweight/day as well as intake per day/kg were large and positive. Back-loin length and rump length, and moreover muzzle width and frame, showed negative correlations with RFI value. However, bulls with better RFI values associated with lower legs score. Results reveal that RFI was shown beneficial correlations with economically relevant self-performance traits. Further investigations are needed to seek additional indicator traits that are predictive for RFI.

## 1. Introduction

Recent increases in the costs of feeds have inspired considerable interest in the use of genetic selection strategies to improve feed efficiency in beef cattle [1]. Thus, breeding programs that can produce animals that require fewer feed inputs without negatively impacting performance traits will improve the profitability of beef cattle production systems [2,3,4].

In the last years, residual feed intake (RFI) has been evaluated as an alternative trait for use in selection programs to improve feed efficiency [5,6]. Residual feed intake, the difference between observed and expected intake, was first proposed by Koch and coworkers [7] who suggested that feed efficiency should be computed as a function of intake and gain over the time. Residual feed intake is calculated using a regression equation involving metabolic body weight and average daily gain, where a more efficient animal will have a negative or low RFI, indicating they consume less than predicted [8]. The conventional basic multiple regression model uses other potential predictors; such as measures of body composition [9,10] which can also be included [11]. Moreover, different statistical approaches were found in literature, so the results are not easily comparable [12]. Recently, Esfandyari and Jensen [13] suggested to avoid the analysis of derived traits as well as the use of a two-step procedure for computing RFI. The new index the residual concentrate intake (RCI) was also defined, and it was calculated as the residuals of the linear regression of concentrate intake on metabolic live weight and average daily gain [14].

For the determination of RFI (or RCI) requires accurate measures of individual animal feed intake. Recent technological advances in feed intake monitoring systems (electronic feed weighing system, automatic feeding system) could be able to recognize individual animals and accurately measure the feed that particular animal consumes. Automated systems have been developed by Calan Broadbent (American Calan Inc. Northwood, NH, USA), Gallagher Animal Management Systems (Gallagher, Hamilton, New Zealand), GrowSafe 4000 System (GrowSafe Systems, Ltd., Airdrie, AB, Canada), the CRFI (BioControl, Technology for biology, Barcelona, Spain), SmartFeed (C-lock Inc., Rapid City, SD, USA) and the RIC-system (Insentec B.V., Marknesse, The Netherlands). The majority of systems currently available are based on variants of RFID technology, with sensors specifically designed to capture the animal’s presence or absence [15], or from an open [16] or gated feed bunk [17,18]. With the increasing availability of this feeding system, it is capable of monitoring individual animal behavioral responses as well [19,20]. Feeding behavior means the amount and distribution of feed intake. First, Nielsen [21] proposed to measure six different parameters: dry matter intake (DMI; kg/day), average intake per visit to the feeder (kg/visit), number of visits to the feeder (visits/day), time spent in the feeder (min/day), average time per visit (min/visit), and feeding rate (g/min). It is well known that feeding behavior is regulated by several internal and external factors, which is important for improving feed efficiency. Nevertheless, feed efficiency has a significant impact on animal performance and animal productivity. More efficient animals use less feed for maintenance, which increases the energy allocated to production (for example, growth). This not only leads to higher economic profitability but also to less waste products (manure, greenhouse gases, etc.) emitted to the environment [22].

In order to further improve accuracy of RFI, the main task is to seek additional indicator traits that are predictive for RFI. The high cost of measuring RFI (or RCI) represents a strong limitation to population-wide selection programs. The genome-wide association study (GWAS) is a useful tool to understand the genetic basis of this trait and to select genes that could be associated with it. In an Italian Brown Swiss population, 11 markers and 48 candidate genes were obtained in GWAS developed for RCI [14].

Meale and coworkers [23] used potential of biomarkers from less invasive samples (blood, hair, and feces) as indicators of feed efficiency, with the central focus on RFI in growing Charolais bulls fed high forage diets. Imaz and coworkers [24] studied the usage of electronic feeders and automatic weighing systems for assessment feeding behavior and growth of Charolais × Angus crossbred weaners in pasture based production system.

Higher DMI has been associated with a lower feed efficiency. Dry matter intake in dairy populations can be predicted with accuracies up to 0.43 and 0.64 by a combination of conformation traits [25]. Kenny and coworkers [11] reviewed a mean R^2^ of 0.70 for the ‘base’ model used to predict dry matter intake in growing (finishing) beef cattle.

These feed-intake-related traits indicate economically relevant information, which can be include into performance test, and its correlations with other related traits (i.e., performance, conformation) are also required to determine. Therefore, the overall aim of this study was to evaluate the effect of differences in residual feed intake of Charolaise candidate young bulls on feeding behavior and self-performance test results and to evaluate relationship of RFI with other available informative traits (feed intake related traits, dry matter intake and conformation) recognized during self-performance test period.

## 2. Materials and Methods

The data of progeny test of Charolaise young bulls were used in this study, and the trial was conducted an experimental farm at Kaposvár Campus of Hungarian University of Agriculture and Life Sciences (MATE) in Hungary. This station evaluated candidate young bulls from February 2021, pre-selected early based on weaning results, morphological criteria, and pedigree. These candidate animals (*n* = 20) came from 10 private farms across from Hungary and were the product of 13 different sires. Experimental procedures and animal care did not specifically impose stressful situations for animals and so institutional animal care and use committee approval was not required under European regulations.

Feed was provided ad libitum from automatic feeders (RIC2, HOKOFARM Group, INSENTEC VB, Markenesse, The Netherlands), which recorded the start and stop weight of the feed alongside time of entry and exit from the individual feeder for each visit by each animal. There were four feeders per pen (2.5 animals/pen) and a water trough providing ad libitum access to water. Two pens were allocated, each pen contained ten animals, and the average weight of animals/pen were balanced. Bulls were 331 ± 29 days of age and 478 ± 55 kg bodyweight (BW) at the start of the experiment. There was a four-week adaptation phase to allow the animals to adapt to the pens, the new social group and the automatic feeders, and to gradually introduce the test diets. The 85-day performance and feed efficiency testing phase started directly afterwards. The 85-day is within the recommended period to determine animal performance without losing accuracy, a major limitation to measuring feed efficiency in nonexperimental settings is the need for strict measurement of individual intake and weight gain over a period of at least 70 d. Animals were weighed on two consecutive days at the beginning, end, and at regular intervals throughout the test period on a calibrated weigh scale.

Diets of bulls were formulated by Vitafort Nutrition Company (Vitafort First Hungarian Feed Production and Distribution Zrt., Dabas, Hungary) such that animals received a diet with increased concentrate and decreased roughage percentages during the test period. Young bulls were fed with TMR an ad libitum basis, which was composed of 32% Vitalbull^®^ concentrate feedstuff (Vitafort First Hungarian Feed Production and Distribution Zrt., Dabas, Hungary), 26% alfalfa hay 21% alfalfa haylage and 21% triticale haylage from day 0 to day 15 (forage to concentrate ratio: 35:65), thereafter forage to concentrate ratio was increased (32:68) in the diet, which contained 35% concentrate feedstuff, 25% alfalfa hay, 20% alfalfa haylage and 20% triticale haylage (Table 1). Feeding was scheduled between 800 and 1000 h a.m. in every day of test period.

Total feed intake of each animal during the feeding period was converted to total DM intake. Daily dry matter intake (DMI: kg/day) was calculated for each animal. Feed conversion ratio for each animal was calculated as the ratio of ADG to DMI (G:F). The residual feed intake was determined as the residual of the regression of DMI versus mid-test metabolic BW (BW 0.75) and average daily gain (ADG) and back fat thickness measured via ultrasound between 12–13th ribs at the end of test period. “Efficient” cattle are those that eat less feed than expected based on their body weight and performance and are termed as having a negative, or low, RFI. “Inefficient” cattle are those that eat more feed than expected based on their body weight and performance. These are termed positive, or high, RFI. High and low RFI groups have been defined as residuals are above > 0.5; (high-RFI, *n* = 10 values from 0.41 to 1.94) or below < 0.5; (low-RFI *n* = 10 values from −2.11 to 0.17), respectively.

Ultrasonic fat depth at the 12th/13th rib as well as rump fat (P8) was taken for all bulls at the end of the 85-day test period. On ultrasound scans longissimus muscle area and intramuscular fat level were evaluated too. The ultrasound measurements were taken with an Aquila Pro diagnostic real-time ultrasound with an 18-cm, 3.5-MHz linear array transducer (Pie Medical Equipment B.V., Maastricht, The Netherlands) using procedures described previously by Török [26].

At the end of the self-performance test, linear scores for muscular, skeletal, and functional abilities were assessed for each animal. The linear scale used is 1–10, where 1 is weak/thin/narrow/shallow/flat/short and 10 is strong/thick/wide/deep/round/long. The score of 5 considered as average value for examined trait, whilst score of 6 means better than average. Muscular traits assessed are shoulder width, back width, roundness of thigh, width of thigh and loin thickness. Skeletal abilities are canon girth, back-loin length, rump length, width of hip bones, and frame. Functional traits assessed are muzzle width, forelegs, hind legs, and top line straightness. Chest depth, chest width, rump width, thigh length, and body condition were also recorded. Body condition score (BCS) of bulls were described through the use of a nine-point scale, where BCS 1 animal is extremely thin while a BCS 9 one is extremely fat and obese [27]. The evaluation was performed by a team of well-trained breeders/technicians of National Association of Hungarian Charolais Cattle Breeders (MCTE) discussing given points one-by one.

Feeding behavior was monitored automatically during performance test period using the HOKO feeders (RIC2, HOKOFARM Group, INSENTEC VB, Markenesse, The Netherlands) which recorded every time each bull entered the feeder providing the number and the duration of feeding events per steer per day. The feeders measured the weight of feed consumed during each visit. The daily dry matter intake (DMI, kg/day), intake g/kg body weight (BW)/day, visits per day, intake per visit (g), time spent eating (min/day), time per visit (min), and eating rate (g/min) were all calculated from data for each individual candidate bulls on a daily basis. Daily intake variation, measured as coefficient of variation (CV, %) and standard deviation for individual animals. Feeding events were then refined by eliminating visits in which no feed was consumed.

Percentage of bulls of group present at the feed bunk, percentage of feed intake and percentage of time spent eating were recorded some time interval of the day the animal consumed the feed. The intervals were broken down as 6:00 to 9:00 a.m., 9:00 to noon, noon to 3:00 p.m., 3:00 to 6:00 p.m., 6:00 to 9:00 p.m., 9:00 to midnight, midnight to 3:00 a.m. and 3:00 to 6:00 a.m.

All statistical procedures were performed SPSS 20.0. Variables (IBM SPSS Statistics for Windows, Version 20.0. IBM Corp, Armonk, NY, USA) were checked for normality using histograms. Performance measurements and feeding behavior were analyzed using generalized linear model including RFI group as a dependent variable. All mean differences were assessed using pairwise comparisons of least squares means. The relationship between individual feeding behavior, performance and conformation traits were evaluated using a Pearson correlation test. An *p* ≤ 0.05 was considered a significant relationship. A tendency was classified as an *p* ≤ 0.10. Stepwise linear regression analysis was conducted to select independent variables in order to estimate RFI.

## 3. Results

Descriptive statistics of performance traits and judgement of skeletal, muscularity and functional traits of candidate young bulls are shown in Table 2 and Table 3.

Candidate young bull performance data during the test period including weight gain, average daily gain (ADG) was similar for groups, the average ADG for low RFI group and high RFI group was 1950 ± 283.5 and 1900 ± 252.5 g/day, respectively (*p* = 0.7). Final live weight of low and high RFI group varied between 615 ± 63 kg and 622 ± 43 kg (*p* = 0.8). At the same time feed intake was affected by groups, low RFI bulls having significantly lower feed/DM intakes (*p* = 0.004) and higher gain to feed ratio (*p* = 0.005).

We observed that residual feed intake of bulls belonging to high RFI group had value of 1.4, but low RFI group had −0.46 (*p* = 0.000). There was no RFI effect on longissimus muscle area (LMA), back fat thickness (BFT) and rump fat thickness (RFT). The LMA was greater, but RFT was lower in H-RFI animals by 6.8 cm^2^ and 0.5 mm, respectively. Similar values were detected for BFT in two groups (L-RFI: 4.9 and H-RFI: 4.4 mm). Concerning marbling, more intramuscular fat level had L-RFI bulls, than other group, however, the differences is not significant.

Effects of RFI group on muscularity and skeletal score was not significant. H-RFI group had higher muscularity score (6.2–6.6, better than average) concerning all examined traits. L-RFI group had greater back loin length and rump length score by 0.5–0.1, respectively. Moreover, L-RFI group had greater frame than H-RFI group.

Concerning functional traits, there were no significant RFI effect except for fore legs (*p* < 0.02) and hind legs (*p* < 0.09). L-RFI group showed lower score for all examined traits. BCS score was better than good (6) at average, L-RFI group had BCS of 6.33, but H-RFI group had BCS of 6.7 (*p* = 0.9).

Traits concerning feeding behavior of L-RFI and H-RFI group are shown in Table 4.

Young bulls with low RFI had lower intake g/body weight/day (*p* < 0.001). Feeding behavior of low RFI group significantly differed (*p* < 0.05) from high RFI animals; they had lower visits number per day (32 vs. 54), but spent longer time per visit (4.5 vs. 2.8 min), and tended to have higher intake g/visit (324 vs. 237 g, *p* = 0.06). A tendency was observed for young bulls in high RFI group to have greater (*p* = 0.08) standard deviation for daily dry matter intake than young bulls with low RFI. No group effects were observed for time spent eating per day and eating rate g/min (*p* > 0.35) as well as CV% of daily DM intake (*p* > 0.5).

**Figure 1:**
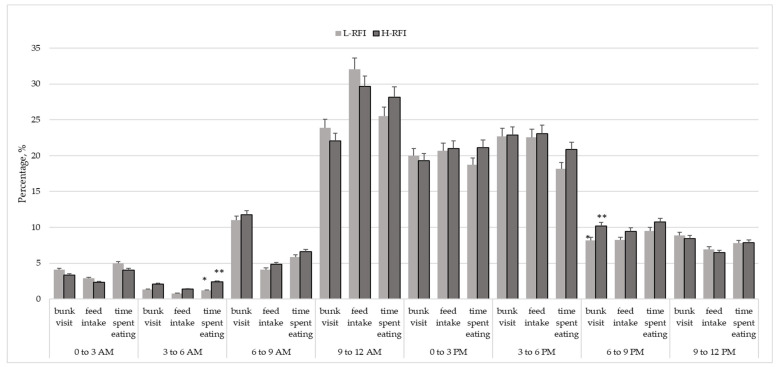
Variation of bunk visit, feed intake and time spent eating of low and high RFI groups. RFI: residual feed intake. (different *, ** asterisks mark showed significant differences (*p* < 0.1) between columns.

The feeding activity was widely spread out over the course of the day with highest percentages of animals present at the feed bunk from 9 a.m. to noon (Figure 1).

Maximum percentage of feed intake and time spent eating also occurred in this time. Low percentages of animals feeding during the night and early morning hours (maximum around 6:00 h). The lowest frequency of bunk visit, feed intake as well as time spent eating were observed around 3:00 and 6:00 h a.m. Moreover, higher percentage of bulls in H-RFI group tended to spent eating in this period. Two further bunk visits period with higher maximum percentages of 43% bulls eating were observed in the afternoon and evening (15:00 and 21:00 h). Higher proportion of bulls in H-RFI group visited feed bunk from 18:00 to 21:00 hours (*p* < 0.09) compared to L-RFI group.

The Pearson correlation coefficients of various variables of feeding behavior and conformation score (muscular, skeletal, and functional traits) to performance data are provided in Table 5.

The correlation coefficient of intake g/body weight/day to RFI was highest (R = 0.9) followed by intake per day/kg DM (R = 0.8), intake g/visit (R = 0.6), forelegs (R = 0.6). Negative associations (R = −0.5–−0.6) were investigated between gain to feed, back loin length, muzzle width and frame as well as time per visit and intake per visit to RFI. RFI was independent of ADG and ultrasound measured traits.

Gain to feed had not significant correlations with conformation traits exception of chest depth (R = −0.4). Correlations of G:F with behavioral traits were not significant. The average daily gain during test positively correlated to G:F (R = 0.7) and time spent eating and intake per day (R = 0.5).

Among ultrasound data, LMA showed some significant positive associations with conformation traits including roundness of thigh (R = 0.8), with of thigh (R = 0.7), shoulder width, loin thickness, rump length, width of hip bones, chest width, rump width (R = 0.6) and both chest depth and thigh length (R = 0.5). Eating rate and intake per day/kg DM also positively correlated to LMA. The back fat thickness of bulls exhibited negative correlation with top line straightness (R = −0.7), whereas positive correlation was observed between back fat thickness and BCS (R = 0.6). Negative correlations were investigated between top line straightness and P8 as well as IMF. Correlations of IMF to eating behavior traits were small and non-significant, except for eating rate (R = 0.4)

The RFI prediction model results using different trait combinations are shown in Table 6. The intake g/bodyweight/day used alone as predictor traits in RFI prediction resulted in good prediction capacity with R^2^ = 0.86 and SEE (standard error of estimate) = 0.416. The intake g/bodyweight/day and gain to feed explained 96% of the variation in RFI with SEE = 0.22. Intake per day, rump fat thickness, chest depth, and thigh length added to predictors traits improved RFI model performance, such that R^2^ increased from 0.98 to 0.99 and SEE decreased from 0.06 to 0.03.

## 4. Discussion

Dry matter intake (DMI) is a primary factor affecting animal performance and the key component to calculate feed efficiency. The practical implementation of individual DMI might be challenging, primarily since individual feed intake records are available in performance test station. According to the results, there were no statistical differences in average daily gain between the two groups. Low RFI animals perform as well as high RFI animals, but significant statistical difference in DMI were detected between the two groups. Our results showed a difference of 14% in DMI between low RFI and high RFI groups, similarly to Fitzsimons and coworkers [28] in finishing Simmental bull. Therefore, it can be considered that low RFI animals are more efficient as their performance is the same, but less feed resource was needed to capture that performance. Animals with low RFI are efficient in feed utilization, as demonstrated by their good production potential despite lower feed intake, without losses in growth or ruminal and metabolic parameters investigated by Trevizan and coworkers [29]. Our results are in agreement with previously reported literature data [30,31,32,33,34,35].

One response to selection for low RFI can be an alteration in body composition against fat deposition [36]. However, this study has shown that RFI had no significant effect on back fat or rump fat thickness. These findings are similar to Consolo and coworkers [31] and Fitzsimons and coworkers’ [28] results, but they found that high RFI animals have a greater content of subcutaneous body fat than those of low RFI. Previously, Baker and coworkers [37], McDonagh and coworkers [38], and McKenna and coworkers [35] also observed no association between body composition and RFI. In one study, low RFI steers had less rump fat and rib fat at the beginning of the study, but no significant difference was observed at the end [39]. Results in the literature [17] showed that RFI had no effect on LD area similarly to our results. In current study, intramuscular fat level of low RFI animals (5.13) similar as high RFI contemporaries (5.06). In another study Perkins and coworkers [40] found that intramuscular fat level was greater in low RFI cattle than high RFI cattle. In a review, Kenny and coworkers [11] confirmed that in terms of relation to muscle accretion, no difference was founded in live animal measures between cattle of high- or low-RFI status. Additionally the relationship between variation in RFI and ultrasonically measured back fat depth again failed to observe a difference. According to their findings, they suggested that RFI rank in growing cattle is not associated with final muscle area, carcass muscle area, and change in back fat depth during the linear phase of the growth curve (typical of RFI test periods in many studies).

Although bulls of low and high RFI in the current study had similar conformation score, which reflects similar muscularity and skeletal traits, high RFI bulls obtained better conformation score except for back loin length, rump length and frame. Basarab and coworkers [9] found that low RFI steers had lighter weights of stomach complex, intestines and liver than high RFI steers, which explain the lower conformation score of the same part of body in our study.

Forelegs and hind legs score is an integral part of bulls’ fertility evaluation. Bulls with undesirable legs score will be culled. Wang and coworkers [41] concluded that fertility traits were not different between bulls categorized as low or high RFI bulls, but feet and legs culling rate varied between 8.61 and 11.66%. In other studies found that L-RFI bulls displayed lower sperm motility, decreased progressive sperm motility, as well as a smaller scrotal circumference [42]. These suggest that some fertility issues may exist with L-RFI bulls.

Body condition score did not differ between groups, but high RFI animals obtained higher score. These finding agree with those of Fitzsimons and coworkers [28].

Concerning eating behavior, the average time spent eating per day in this study was 133 min/day, similar to Haskell and coworkers [43] carried out experiment with Charolaise steer. In our study where stocking density was approximately 2.5 animals per feed bunk, and feeding time was affected by RFI group. Our results showed that low RFI cattle spent, on average, 1.63 min longer eating, out of an average of 7.66 min within a 24-h period, then their high RFI contemporaries. So, they spend longer time eating, resulting improvement in feed digestibility due to an increased production of saliva. As a consequence of this, a positive effect on feed efficiency was observed. Contrary to Trevizan and coworkers [29], our results showed that low RFI animals spent less time at the feed bunk when compared to animals with medium and high RFI for a similar amount of feed consumed per visit. In previous mentioned study, the stocking density (5.9 animals/feeder) was much higher in our study suggesting that feeding time may be affected by group size as well. In the current study, low RFI bulls eat higher amount of feed per visit and had lower visit numbers per day. Similarly to Romanzin and coworkers [44] we found that L-RFI bulls had a lower number of feeding event and same feeding time compared to H-RFI bulls. Eating rates of groups indicated that low RFI candidate bulls have a slower eating rate than their high RFI counterparts. Similar findings described by Montanholi and coworkers [45] for steers. As previously Cantalapiedra-Hijar and coworkers [46] stated the higher dry matter intake of the high RFI animals in that analysis implies that they also had a faster eating rate than the low RFI animals.

Similarly to previous findings [8], low RFI cattle has been shown a reduction in feed intake (intake g/body weight/day), improved feed conversion with no negative effect on body weight or growth. Moreover, Jiu and coworkers [34] found that low RFI beef cattle showed any adverse effect on meat quality and palatability.

The degree of day-to-day fluctuation in dry matter intake (SD of intake per day) had impact on RFI status of animal, it seems so that reducing standard deviation of day-to-day fluctuation in dry matter intake improve feed efficiency of animals. As Pereira and coworkers [47] previously stated, Bos indicus bulls from low dry matter intake fluctuation group improved feedlot performance.

The usage of automatic feeding systems in cattle offers multiple advantages, mostly due to the possibility of an increased feeding frequency [48]. Fattening cattle are commonly fed twice per day, namely in the morning and in the evening, or even only once in the morning using an ad libitum feeding regimen. On pasture, cattle spend about 10–12 h per day grazing, divided into several meals spread out from dusk to dawn. The feeding duration of housed cattle is reduced to 4–7 h per day, but feeding is still divided into 6–12 daily meals spread out over the daylight period [49]. In the present study, we spread out their feeding behavior over the course of the day with reduced feeding activity during the night and early hours of the morning. The feeding activity in the morning during and after the first feed delivery of the day was quite low, indicating that the bulls accumulated sufficient quantities of feed to digest during the night. Bunk attendance of high RFI cattle was more frequent from 9 to 12 a.m. However, they ate a lower percentage and spent less time with feeding than low RFI cattle. Feed efficiency may be influenced by altering physical activity through feeding behaviors [50].

Feeding events per day was positively correlated with RFI, which indicates that efficient animals utilize less energy is by spending fewer time (3–9 a.m.) with eating and by lower percentage of bunk visit (6–9 p.m.).

Pearson’s correlations between feeding behavior and RFI showed that intake per day, SD of intake per day, and visits per day were positively correlated to RFI. Conversely, intake per visit and time per visit were negatively correlated with RFI. Similarly, to our findings, DelCurto-Wyffels and coworkers [51] found intake per day was negatively associated to G:F and positively related to ADG. Regarding ultrasonic measurements and body condition score, they were not correlated with RFI [28]. These results show body composition of low RFI bulls are not adversely affected, as compared to high RFI bulls. Thus, selecting animals based on RFI is unlikely to result in an undesirable response in performance traits in growing animals. In general, high RFI is associated with shorter back loin length and smaller frame, and moreover more correct foot structure, contrary to low RFI bulls which had longer back loin length, higher frame, and weaker foot structure. Cattle with larger frame and carcass weight divert energy towards production more efficiently than smaller framed animals that divert more energy towards maintenance [52]. Manzanilla-Pech and coworkers [25] found positive genetic association between conformation traits (stature, chest width, body depth) and feed intake (dry matter intake) of dairy cattle. The highest estimated genetic correlations involving dry matter intake were with chest width followed by stature and body depth. In the current study, we also confirmed the positive phenotypic relationship (0.3) between RFI and chest depth as well as chest width of Charolaise candidate bulls. Clauss and Hümmel [53] reviewed that muzzle width variance in ruminants provide selection tool for wider muzzles to enhance foraging efficiency. Based on our data, it can be stated that non efficient bulls (high RFI) had a more narrow muzzle than their efficient contemporaries.

Residual feed intake is a common measure of feed efficiency. However, in practice it is estimated by several ways. The conventional method for the calculation of RFI faces some limitations. In Denmark, potential breeding bulls were tested for growth and feed efficiency performance. Feed intake records were averaged over each weighing period so that each individual record consisted of average weight and average daily feed intake in the period studied [13].

In our study, the multiple regression model was used to predict RFI based on individual feed intake data during the self-performance test period of Charolaise bulls. The experiment reflects the results of McKenna and coworkers [35], who accounted for 70% of the variation in dry matter intake in Simmental cattle. Romansin and coworkers [44] also used multiple regression to predict DMI based on performance data used for RFI calculations and behavioral traits; the base model explained 50.5% of the DMI variability, and an additional 5% of the variation in DMI can be explained feeding behavioral traits. In our study predictor traits among automatic feeding recording data available for RFI were the following: intake g/body weight/day and gain to feed, as well as intake per day. In the base of data delivered from automatic feeding system accounts for up to 98% of RFI model. The prediction models using conformation trait combinations can be further improved.

## 5. Conclusions

Our findings reveal that selecting animals based on RFI does not result in an undesirable response in self-performance traits in Charolaise candidate young bull. Skeletal traits, including back-loin length and rump length (as well as frame) showed negative correlations with RFI value, indicating that the longer, taller, higher frame size bulls were more efficient. At the same time, bulls with better RFI values were associated with forelegs weakness. The close relationship of dry matter intake (DMI) and efficiency in Charolaise candidate bulls suggests that measures of individual DMI are relevant, and may have application to the self-performance test. Further investigations are needed to seek additional indicator traits that are predictive for RFI.

## Figures and Tables

**Table 1:** Ingredients and chemical composition related to dry matter [DM] as well as energy [MJ/kg; %DM basis] of the total mixed ration.

Ingredient [%]	I. Period	II. Period
Vitalbull^®^concentrate feedstuff	32	35
alfalfa hay	26	25
alfalfa haylage	21	20
triticale haylage	21	20
Chemical composition		
Dry matter [%]	69.52	70.52
Crude ash	9.22	9.16
Energy dependent metabolizable protein	9.55	9.64
Crude fat	3.03	3.13
Crude fiber	19.98	19.06
Starch	18.82	20.57
Acid detergent fiber	23.57	22.56
Neutral detergent fiber	33.37	33.30
NEm [MJ/kg]	6.07	6.17
NEg [MJ/kg]	3.43	3.54
Minerals, vitamins		
Ca [g]	0.92	0.92
P [g]	0.35	0.37
Na [g]	0.19	0.21
Vitamin A [IU]	9642.32	10,686.90
Vitamin D [IU]	1902.95	2109.10
Vitamin E [mg]	48.00	53.20

**Table 2:** Performance traits of animals with low (L-RFI) and high RFI (H-RFI).

Traits	L-RFI	H-RFI	SEM	*p*-Value (RFI)
Residual feed intake (kg/day)	−0.64	1.13	0.244	0.000
Dry matter intake (kg/day)	13.24	15.02	0.331	0.004
Body weight, kg				
0 day	453.67	464.90	10.62	0.612
29 day	513.22	522.18	11.13	0.700
57 day	566.67	575.09	11.44	0.725
Final body weight	615.11	622.36	11.53	0.764
Average daily gain (kg/day)	1.95	1.90	0.06	0.693
Total gain (kg)	161.44	157.46	4.84	0.693
Live weight gain	1.53	1.50	0.024	0.808
Gain to feed	0.14	0.12	0.004	0.005
Ultrasonography measurements				
Longissimus muscle area (cm^2^)	86.83	93.61	2.33	0.152
Back fat (mm)	4.9	4.4	0.32	0.974
Rump fat (mm)	6.0	5.5	0.28	0.316
Intramuscular fat level (%)	5.13	5.06	0.26	0.265

**Table 3:** Judgement of animals with low (L-RFI) and high RFI (H-RFI). RFI: residual feed intake.

Traits	L-RFI	H-RFI	SEM	*p*-Value (RFI)
Muscularity (score)				
shoulder width	6.11	6.55	0.233	0.367
back width	5.89	6.36	0.233	0.323
roundness of thigh	5.33	6.18	0.268	0.117
width of thigh	5.44	6.36	0.312	0.147
loin thickness	5.89	6.36	0.221	0.297
Skeletal measurements (score)				
canon girth	4.56	4.91	0.123	0.158
back-loin length	6.55	6.09	0.391	0.569
rump length	5.89	5.82	0.357	0.925
width of hip bones	5.22	6.09	0.272	0.115
frame	5.89	5.27	0.366	0.417
Functional traits (score)				
muzzle width	5.56	5.82	0.300	0.675
forelegs	4	5.45	0.329	0.023
hind legs	4.33	5.5	0.284	0.09
top line straightness	6.11	6.27	0.213	0.716
chest depth	5.33	5.82	0.197	0.231
chest width	5.22	5.73	0.199	0.215
rump width	5.22	6.09	0.272	0.115
thigh length	5.0	5.36	0.258	0.497
Body condition score	6.33	6.7	0.193	0.881

**Table 4:** Feeding behavior of animals with low and high RFI. RFI: residual feed intake; SD: standard deviation; CV: coefficient of variation; DM: dry matter.

Traits	L-RFI	H-RFI	SEM	*p*-Value (RFI)
Time spent eating, per day, min	136.83	129.17	6.04	0.543
Visits per day	32.30	54.19	4.82	0.019
Time/visit min	4.46	2.83	0.353	0.018
Eating rate g/min	146.36	174.59	10.20	0.175
Intake g/body weight/day	21.60	24.17	0.418	0.000
Intake g/visit	324.24	236.97	23.06	0.060
SD of intake per day/kg DM	2.24	2.78	0.150	0.072
CV of intake per day/kg DM	17.12	18.41	0.907	0.494

**Table 5:** Pearson correlation coefficients for pair-wise associations between performance traits, conformation score, and feeding behavior. RFI: residual feed intake; G:F: feed conversion ratio; ADG: average daily gain; LMA: longissmus muscle area; BFT: back fat thickness; P8: rump fat thickness; IMF: intramuscular fat; BCS: body condition score; SD: standard deviation; DM: dry matter.

	RFI	G:F	ADG	LMA	BFT	P8	IMF
G:F	−0.61 **	1	0.67 **	−0.42	-	0.29	−0.42
Shoulder width	-	−0.33	-	0.61 **	-	-	-
Back width	-	−0.27	-	0.53 *	-	−0.26	-
Roundness of thigh	0.29	−0.42	-	0.79 **	-	−0.26	-
Width of thigh	0.21	−0.39	-	0.66 **	−0.2	-	-
Loin thickness	-	−0.21	-	0.57 **	−0.2	-	-
Canon girth	0.28	-	0.24	0.33	−0.2	-	-
Back loin length	−0.45 *	-	-	0.31	−0.32	−0.33	-
Rump length	−0.39	-	-	0.61 **		−0.36	
Width of hip bones	0.26	−0.37	-	0.61 **	-	-	-
Frame	−0.47 *	0.29	0.21	0.25	−0.36	−0.29	-
Muzzle width	−0.50 *	−0.26	-	0.43	−0.22	-	-
Forelegs	0.56 **	0.23	-	0.30	−0.33	−0.23	-
Hind legs	0.34	-	0.32	-	−0.35	-	-
Top line straightness	-	-	-	-	−0.66 **	−0.44 *	−0.46 *
Chest depth	0.29	−0.44 *	-	0.52 *	-	-	-
Chest width	0.30	−0.43	-	0.63 **	-	-	0.21
Rump width	0.26	−0.37	-	0.61 **	-	-	-
Thigh length	-	−0.36	−0.33	0.50 *	-	-	-
BCS	0.20	-	0.26	0.29	0.56 *	0.30	0.27
Time spent eating, per day, min	-	0.21	0.46 *	−0.34	-	0.20	0.35
Visits per day	0.63 **	-	-	-	-	-	-
Time/visit min	−0.56 **	-	−0.22	-	-	-	-
Eating rate g/min	-	−0.23	-	0.44 *	-	-	0.44 *
Intake per day/kg DM	0.75 **	−0.31	0.46 *	0.57 **	-	-	0.38
Intake g/body weight/day	0.93 **	−0.32	-	-	-	0.23	-
Intake g/visit	−0.51 *	−0.22		0.26	-	-	0.22
SD of intake per day/kg DM	0.41	-	0.35	-	-	-	-

* *p* ≤ 0.05; ** *p* ≤ 0.01.

**Table 6:** Linear regression fitting statistics for RFI. RFI: residual feed intake; G:F: feed conversion ratio; P8: rump fat thickness; SEE: standard error of estimate.

Predictor Trait	Factor	Adjusted R^2^	*p*-Value	SEE	Equations
Intake g/BW/day(A)	1	0.86	0.000	0.416	−12.156 + 0.543A
Intake g/BW/day; G:F(B)	2	0.96	0.000	0.220	−7.812 + 0.476A − 21.437B
Intake g/BW/day; G:F; intake per day(C);	3	0.98	0.000	0.139	−8.601 + 0.408A − 19.491B + 0.001C
Intake g/BW/day; G:F; intake per day; P8(D);	4	0.99	0.000	0.064	−9.005 + 0.433A − 16.370B + 0.001C − 1.036D
Intake g/BW/day; G:F; intake per day; P8; chest depth(F)	5	0.99	0.000	0.052	−9.409 + 0.442A − 15.327B + 0.001C − 1.053D + 0.049F
Intake g/BW/day; G:F; intake per day; P8; chest depth; thigh length(G)	6	0.99	0.000	0.037	−9.042 + 0.435A − 16.152B + 0.001C − 0.990D + 0.087F − 0.044G

## Data Availability

The data presented in this study are available on request from the corresponding author.
